# Structural and Optical Characterization of g-C_3_N_4_ Nanosheet Integrated PVC/PVP Polymer Nanocomposites

**DOI:** 10.3390/polym15040871

**Published:** 2023-02-09

**Authors:** Alhulw H. Alshammari, Khulaif Alshammari, Majed Alshammari, Taha Abdel Mohaymen Taha

**Affiliations:** Physics Department, College of Science, Jouf University, Sakaka P.O. Box 2014, Saudi Arabia

**Keywords:** PVC/PVP blend, g-C_3_N_4_, refractive index, optical susceptibility

## Abstract

The present work considers the integration of g-C_3_N_4_ nanosheets into PVC/PVP polymer nanocomposites at ratios of 0.0, 0.3, 0.6, and 1.0 wt%. The XRD data scans showed semicrystalline structures for all PVC/PVP/g-C_3_N_4_ polymer blend films. The FTIR and Raman measurements revealed intermolecular hydrogen bonding between the g-C_3_N_4_ surface and the OH^−^ groups of the PVC/PVP network. ESEM morphology analysis for PVC/PVP/g-C_3_N_4_ nanocomposite films displayed homogeneous surface textures. The data of TGA showed improved thermal stability as the decomposition temperature increased from 262 to 276 °C with the content of g-C_3_N_4_ (0.0–1.0 wt%). The optical absorbance data for PVC/PVP films improved after the addition of g-C_3_N_4_. The optical energy gaps showed compositional dependence on the g-C_3_N_4_ content, which changed from 5.23 to 5.34 eV at indirect allowed transitions. The refractive index for these blend films enhanced (1.83–3.96) with the inclusion of g-C_3_N_4_. Moreover, the optical susceptibility for these nanocomposite films increased as the content of g-C_3_N_4_ changed from 0.0 to 1.0 wt%. Finally, the values of the nonlinear refractive index showed improvement with the increased percentage of g-C_3_N_4_. When g-C_3_N_4_ was added up to 1.0 wt%, the DC conductivity improved from 4.21 × 10^−8^ to 1.78 × 10^−6^ S/cm. The outcomes of this study prove the suitable application of PVC/PVP/g-C_3_N_4_ in optoelectronic fiber sensors.

## 1. Introduction

Polymers have been widely used in various applications due to outstanding properties such as their low cost, stability, easy fabrication, etc. The advantages of polymer matrix composites include their low cost and straightforward fabrication processes. Furthermore, polymer composites can be used as the primary material to create lightweight, flexible electronics, which is advantageous considering consumer demand. There are different types of polymers, for example, polyvinyl chloride (PVC) and polyvinylpyrrolidone (PVP). Moreover, polymers have different optical properties which are an essential factor to be investigated since these polymers have wide applications, i.e., energy, photonic, and optoelectronics [1,2,3]. In comparison to polymer composites using traditional micron-scale fillers such as carbon fibers or glass, polymer nanocomposites display significant property improvements at considerably lower filler loadings, which can lead to a significantly reduced component weight and simplify manufacturing. Individual nanoparticles homogeneously dispersed in a matrix polymer help compensate the ideal nanocomposite design. The dispersion state of nanoparticles determines the enhancement of properties to their greatest potential. A significant interfacial area between the components of the nanocomposites may result from the homogeneous dispersion of the nanofillers. Several parameters, including polymer matrix properties, nature and type of nanofiller, polymer and filler concentration, particle size, and particle distribution, are believed to contribute to the enhanced action of the filler [4].

A variety of procedures are performed on polymeric materials during polymer processing to maximize their utility. As the properties of polymers differ from one type to another, producing a blend of two polymers within a suitable ratio may lead to a polymer blend with combined properties which do not exist in a single polymer. Polymer blends have been used in various applications such as PVA/PVP/Ag film coating [5], PMMA/PVP/BaTiO_3_ for optoelectronics [6], and PMMA-PCL-gelatin for biomedical applications [7]. The preparation of a PVC and PVP blend has been reported where PVC acted as a proton donor and PVP with its carbonyl group as a proton acceptor [8]. Doping the polymer blends with appropriate nanosheets is a significant avenue toward the achievement of polymer nanocomposite blends with high performance, because nanoparticles can enhance their physical, chemical, and mechanical properties. Nanosheets have increased mechanical strength, an extremely thin structure, and a high surface-to-volume ratio. They demonstrate improved hydrophobic contact, physical adsorption, van der Waals force, and electrostatic attraction with polymers. In addition, nanoparticles are utilized to reinforce the polymer blends by overcoming their limitations [9], and the selection of these nanosheets as a dopant is controlled by the desirable application. The nanoparticle and polymer PVC/PVP blends are often subjected to hydrogen bonds through the hydroxyl group of PVP and nanoparticle surfaces [10]. Hydrogen bonds cause the enhancement in the optical and thermal properties of polymers nanocomposites [11]. Changing the optical characteristics of polymer materials is one of the most significant consequences of nanoparticles. The full integration of nanosheets with polymers is required for enhanced electron transport between nanosheets and polymers, particularly in electronics applications. For applications such as micro-optics and optical data transmission, changing the refractive index of polymers is particularly crucial [5,6].

When nanosheets or other nanomaterials are added to the polymer matrix, the properties of the polymers can be changed, or frequently, new qualities are added. Nanosheets can either be produced inside the polymer matrix or synthesized previously and integrated to the polymer for homogeneous composite films. Doping the polymer blends with low percentages of nanoparticles is favorable due to excellent influences compared with conventional composites [12]. In this light, the optical property of the PVC/PVP blend was enhanced when doped with SrTiO_3_ [11]. A previous study reported how the ZnFe_2_O_4_ nanoparticles affected the physical properties of similar polymer blends [13].

Graphitic carbon nitride (g-N_3_C_4_) has a narrow band gap of 2.7 eV; therefore, it has good light absorption in the visible region [14]. Graphite and g-C_3_N_4_ have comparable but distinct structural similarities. One by one, nitrogen and carbon atoms comprise the hexatomic ring. Every carbon atom forms a large planar network structure owing to covalent bonding with three nitrogen atoms. The g-C_3_N_4_ is a common kind of carbon nitride that has been extensively utilized in catalysis [15,16,17]. Graphitic carbon nitride is a well-known organic semiconductor with distinct electronic, optical, and mechanical properties. Therefore, g-N_3_C_4_ has high potential to be used in optoelectronic applications, as reported in the literature [18,19]. In the literature, the advantage of using g-N_3_C_4_ as a polymer dopant was reported. For example, the incorporation of g-N_3_C_4_ into the PVA polymer film enhanced its thermal conductivity and electrical insulation [20]. Thus, integrating the polymer blends with such material has high potential to enhance their optical properties. To the best of my knowledge, in the literature, only a few studies have reported on polymers doped with graphitic carbon nitride for optoelectronic applications, leaving a research gap to be studied; therefore, in this work, we concentrate on that aspect.

One of the earliest methods for fabricating polymer films was solution casting. Later, extrusion, pressing, and polymer blowing from the melt assumed the function of this technique. However, high-quality thin-film structures with enhanced optical and physical features are produced by solution casting. The finished product has benefits including features such as dimensional stability and thickness homogeneity. This method requires the low temperature of synthesis. Moreover, this method possesses a low-cost, easy-to-control post-casting drying time and a simple procedure of preparation [21,22,23].

Herein, PVC/PVP polymer blends doped with various g-N_3_C_4_ percentages (0.0–1.0 wt%) were prepared via solution casting. The polymer blends’ nanocomposites were characterized by advanced techniques, i.e., XRD, Raman, FTIR, SEM, TGA, and optical absorption as well as dielectric measurements. We determined the linear and nonlinear optical parameters of this polymer blend’s nanocomposites when increasing the g-N_3_C_4_ proportions.

## 2. Experimental Section

The g-C_3_N_4_ nanosheets were prepared from high-purity urea (MERK, Darmstadt, Germany) by the polycondensation method at 500 °C at a heating rate of 3.0 °C per minute, and the process took 2.0 h [24]. The product was collected and ground well inside an agate mortar. The PVC/PVP polymer blend was developed using analar-grade PVC (MERK, Darmstadt, Germany) and PVP (LOBACEMIE, Mumbai, India) polymer powders. Moreover, the solvent tetrahydrofuran (THF) was supplied from CARLO ERBA (Cornaredo, Italy). For the preparation of PVC/PVP/g-C_3_N_4_ nanocomosite films, 0.9 g of PVC powder was dissolved in 30 mL of THF for 60 min at 300 K. In addition, 0.1 g of PVP powder was dissolved in 5.0 mL of THF and added to the PVC clear solution. The PVC/PVP mixture was stirred for another 60 min with the addition of 0.0, 0.1, 0.3, 0.6, and 1.0 wt% of g-C_3_N_4_ nanosheets. The solution mixture was subjected to ultrasonic waves for 30 min. Finally, the PVC/PVP/g-C_3_N_4_ nanocomposite solution was poured inside a polypropylene dish. The nanocomposite films were collected after drying inside an electric oven at 50 °C for 48 h.

The Shimadzu XRD 7000 (Kyoto, Japan) diffractometer produced the crystal structure data for the PVC/PVP/g-C_3_N_4_ nanocomposite films. The Shimadzu FTIR–Tracer 100 spectrometer (Kyoto, Japan) recorded the transmittance vs. wavenumber for the PVC/PVP/g-C_3_N_4_ blend films. Quattro environmental scanning electron microscope (ESEM) produced surface morphology scans of the PVC/PVP/g-C_3_N_4_ nanocomposite films. A Shimadzu TGA-51 thermogravimetric (Kyoto, Japan) analyzer was used for TGA measurements at temperatures 30–600 °C, and the heating rate was 10 °C/min. The Cary 60 UV-Vis spectrophotometer recorded the optical absorption data for the PVC/PVP/g-C_3_N_4_ films. The DC conductivity data for the polymer films were completed on two-probe setup at 300 K on an MTZ-35 impedance analyzer.

## 3. Results and Discussion

**Figure 1:**
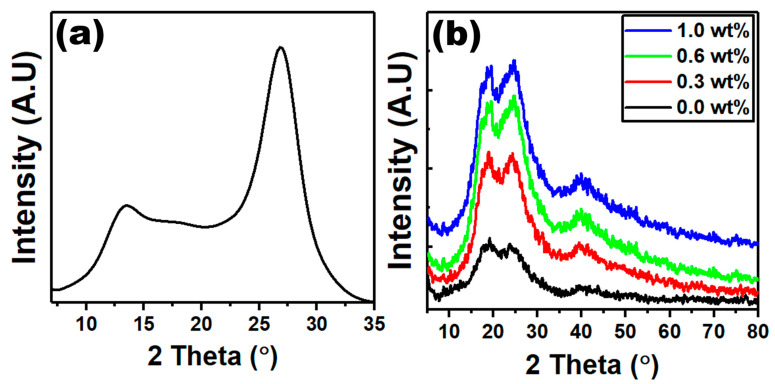
XRD diffraction patterns of (**a**) g-C_3_N_4_, (**b**) XRD diffraction patterns of PVC/PVP doped with different concentrations of g-C_3_N_4_.

One of the most effective non-destructive methods for determining the crystallographic structure of polymer nanocomposites is X-ray diffraction. X-ray diffraction (XRD) was used to characterize the polymer PVC/PVP doped with nanosheet g-C_3_N_4_, as shown in Figure 1. The highest diffraction peak of g-C_3_N_4_ (002) is located at around 27.4° (Figure 1a), which is consistent with the stacking peak of interplanar aromatic systems [25,26], while the reflection peak (100) located at 13.2° is consistent with the inter-layer structure packing of the lattice planes [27]. The XRD data reveal the successful synthesis of the g-C_3_N_4_ nanosheet. Due to the small amount of g-C_3_N_4_ in the polymer composites, the diffraction peaks of g-C_3_N_4_ were not observed in the XRD spectra. Two diffuse peaks at 18.0° and 24.0° in the spectra of PVC/PVP (Figure 1b) reflect the semicrystalline nature. Additionally, there is a slight shift toward a higher angle in the peak position, which is assigned to the strong interaction between the PVC/PVP polymer blend and the g-C_3_N_4_ nanosheet.

The Segal equation was used to calculate the crystallinity index (*CI*) for PVC/PVP/g-C_3_N_4_ nanocomposites, as described below [28]:

(1) $$CI=\frac{I_{24}-I_{18}}{I_{24}}\times \;100$$

where *I*_24_ represents the intensity of the peak at 2θ = 24°, and *I*_18_ at 2θ = 18°. Accordingly, the crystallinity index data for PVC/PVP films are 1.01, 1.20, 3.0, and 4.26 at 0.0, 0.3, 0.6, and 1.0 wt% of g-C_3_N_4_, respectively. From these data, a slight increase in *CI* was due to the small content of g-C_3_N_4_. The full width at half maximum (*β*) of a diffraction peak is inversely proportional to the crystallite size (*D*), as explained by the Scherer equation [29]:

(2) $$D=\frac{0.9\lambda }{\beta \cos\theta }$$

where the X-ray wavelength (*λ* = 1.54056 Å). As seen in Figure 1b, the peak broadening of PVC/PVP increased with the gradual increase in g-C_3_N_4_ content. This, in accordance, led to a decrease in crystallite size.

**Figure 2:**
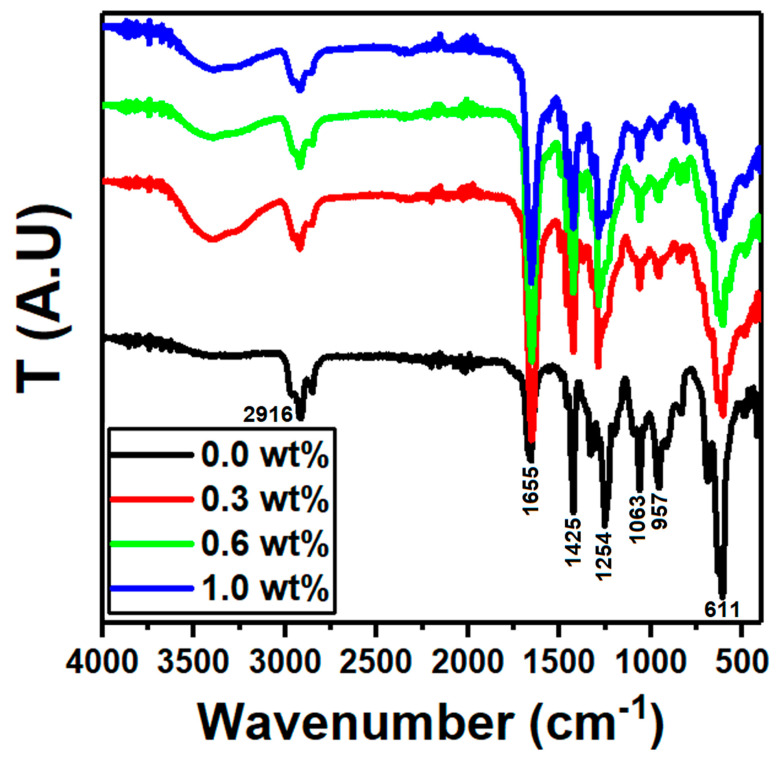
FTIR spectra for the PVC/PVP/g-C_3_N_4_ nanocomposite films.

An efficient method for characterizing polymeric materials is Fourier transform infrared spectroscopy (FTIR). A unique fingerprint is recognized for a sample concerning the measured spectrum. The FTIR spectra of PVC/PVP/g-C_3_N_4_ nanocomposites at different concentrations of nanosheet g-C_3_N_4_ are shown in Figure 2. The presence of the vibrational frequencies of pure PVC/PVP polymers in the spectrum of the produced blend indicates the formation of hydrogen bonds. PVP and other polymers with electronegative oxygen and ternary amide groups have the potential to be effective proton acceptors due to the nature of the basic functional groups. It is assumed that the interaction will consist of hydrogen bonds between the C–H of CH_2_ in PVC and the C–O in PVP. A broad absorptions peak at 3370–3420 cm^−1^ is ascribed to hydroxyl group O–H stretch vibrations, while a sharp absorptions peak at around 1655 cm^−1^ is assigned to O–H stretch bending vibrations, respectively [30]. The observed peaks at 1324 and 1425 cm^−1^ are attributed to C-O and C=C vibrations, respectively [31]. The bands at around 692, 833, and 957 cm^−1^ correspond to C-H bending vibration out-of-plane [31,32]. The bending and stretching vibrations of C-N correspond to the formation of the absorption bands at 1425 and 1290 cm^−1^, while the C-Cl bonds are ascribed to the bands at 611 and 636 cm^−1^ [33]. As the concentration of g-C_3_N_4_ wt% increases, the position of the broad peak in the pure PVA/PVP spectrum shifts from 3370 to 3420 cm^−1^. Additionally, the characteristic peak at 1254 cm^−1^ related to C-N bending vibrations of the pure PVA/PVP shifts to 1290 cm^−1^. These variations in peak positions are attributed to the intermolecular hydrogen bonding between the g-C_3_N_4_ surface and the -OH groups of PVC/PVP, which indicates the success of the interaction between g-C_3_N_4_ nanosheets and PVC/PVP polymers. The distribution of potential energy throughout the host polymeric blend’s chains is significantly impacted by this interaction, which also modifies the backbone structure of the blend [34]. This g-C_3_N_4_ filling-induced structural modification of the polymeric blend’s chains significantly affects the physical properties of the g-C_3_N_4_-filled polymeric nanocomposites [35].

**Figure 3:**
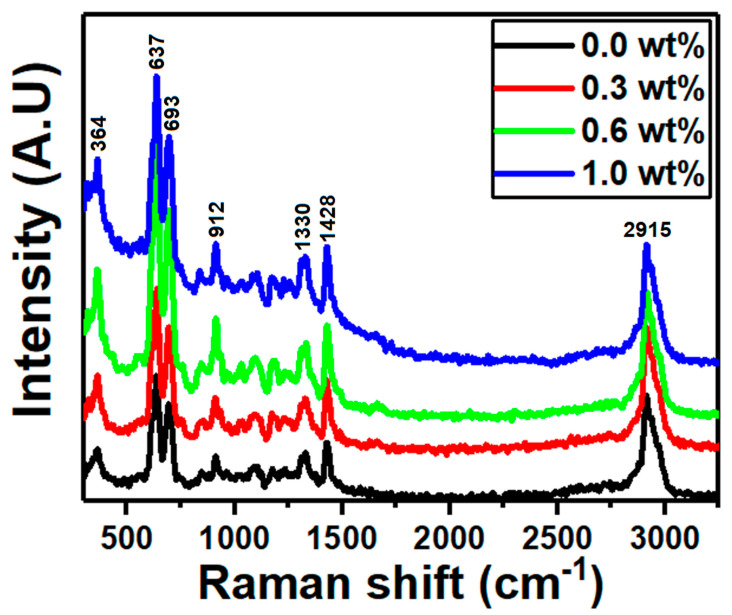
Raman spectra for the PVC/PVP/g-C_3_N_4_ nanocomposite films.

The vibrational, rotational, and other low-frequency modes recorded by Raman spectroscopy allow us to assess the structural properties of materials. Thus, Raman scattering is used in Raman spectroscopy to identify molecular vibrations. The Raman spectra for polymer PVC/PVP doped with different concentrations of g-C_3_N_4_ are shown in Figure 3. The stretching vibration of C-Cl bonds causes the high-intensity peaks at 637 and 693 cm^−1^. Additionally, the intensity peak at 2915 cm^−1^ is the result of the stretching vibration of the C-H and C-H_2_ bonds [36]. The stretching vibrations of C=C bonds in conjugated aromatic composites are ascribed to the bands at around 1428 cm^−1^, while the defect structure of carbon material is related to the bands around 1330 cm^−1^ [37].

Due to the asymmetric stretching vibration, this intensity of quenching validates the interaction between polymer PVC/PVP and nanosheet g-C_3_N_4_ in the C-Cl region [11].

**Figure 4:**
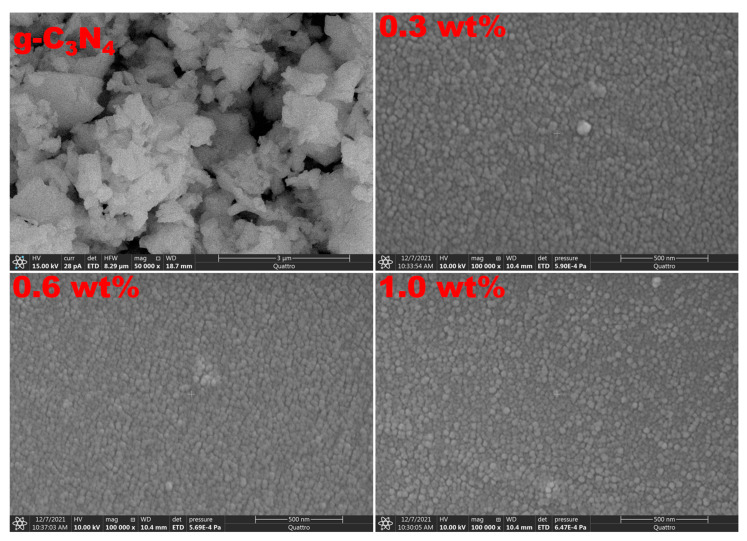
ESEM micrograph surface morphology scans for the PVC/PVP/g-C_3_N_4_ nanocomposite films.

In comparison to an optical microscope, scanning electron microscopy is frequently employed to analyze the sample surface morphology. ESEM images were used to examine the morphology and structure of the synthesized g-C_3_N_4_ nanosheets. The 2D sheet structure shown in Figure 4 is consistent with reports for g-C_3_N_4_ materials. Moreover, the image shows that g-C_3_N_4_ is stacked flakes. The ESEM surface morphology analyses for the PVC/PVP/g-C_3_N_4_ nanocomposite films are displayed in Figure 4. All the polymer films showed homogeneous surface morphology and interconnection between g-C_3_N_4_ and the PVC/PVP blend network.

**Figure 5:**
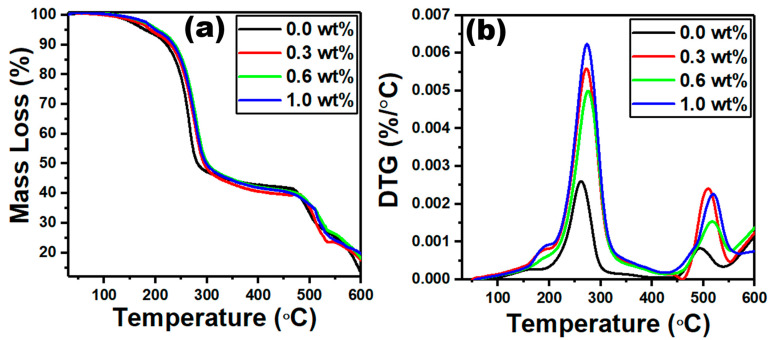
TGA (**a**) and DTG (**b**) graphs for the PVC/PVP/g-C_3_N_4_ nanocomposite films.

Thermogravimetric analysis (TGA) was used to characterize the thermophysical properties and examine the thermal stability of polymer films. Moreover, TGA is used to measure thermal stability of a sample, the amount of solvent still present, and the amount of water absorbed. This technique measures the mass changes in a sample as a function of time and/or temperature. The thermal stability of a copolymer is typically between that of two homopolymers and varies depending on the copolymer composition. Moreover, TGA analysis has also been used to investigate the effect of filler on a polymeric sample’s thermal stability. The TGA and DTG graphs for PVC/PVP/g-C_3_N_4_ nanocomposites are displayed in Figure 5a,b. The data describing the mass loss vs. temperature shown in Figure 5a revealed a main degradation stage between 180 and 316 °C, which corresponds to the dehydrochlorination of the PVC/PVP polymer blend [38]. The maximum decomposition temperature correlated with this stage was determined from Figure 5b, which shifted from 262 to 276 °C as the content of g-C_3_N_4_ changed from 0.0 to 1.0 wt%. Therefore, the increase in g content resists the dehydrochlorination of the PVC/PVP polymer blend and improves the thermal stability.

A second degradative stage observed at temperatures higher than 400 °C belongs to the scission of covalent bonds in PVC/PVP chains [39]. The decomposition temperature at this stage increased from 509 to 520 °C when the content of g-C_3_N_4_ increased from 0.0 to 1.0 wt%. Moreover, the residual mass % at 600 °C were 13.14, 17.50, 18.12, and 19.71% for the content of g-C_3_N_4_ (0.0, 0.3, 0.6 and 1.0 wt%). All these outcomes prove the enhanced thermal stability of PVC/PVP/g-C_3_N_4_ nanocomposites.

**Figure 6:**
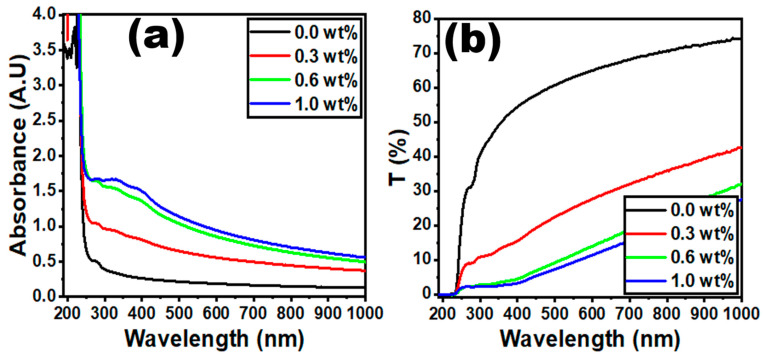
Graphs of (**a**) absorbance against wavelength and (**b**) transmittance against wavelength for the PVC/PVP/g-C_3_N_4_ nanocomposite films.

The absorption spectrum of a material is determined using ultraviolet–visible (UV-Vis) spectroscopy in the specified spectral band. This method is especially beneficial for polymers, because both the π-electrons and non-bonding electrons (n-electrons) enclosed in the molecules can absorb from the ultraviolet to visible ranges at the spectrum energy. In this section, we have studied the optical properties of the PVC/PVP polymer blend matrix doped with g-C_3_N_4_ concentrations of 0.0, 0.3, 0.6, and 1.0 wt%. The absorbance and optical transmittance curves of PVC/PVP/g-C_3_N_4_ nanocomposites have been investigated. Figure 6a represents the absorbance band of PVC/PVP polymer that appears at 280 nm; this could be from the transitions of two unsaturated bonds, C=O and C=C [40,41]. It is notable that a small shift appeared at the absorption band, which is regarded as a redshift. This redshift of absorption bands increases with increasing g-C_3_N_4_ concentrations. Figure 6b shows that the optical transmissions gradually reduce with increasing g-C_3_N_4_ concentrations. The decrease in optical transmissions may be explained by an increase in the photon scattering due to the dense nanoparticles of the polymer blend [14].

**Figure 7:**
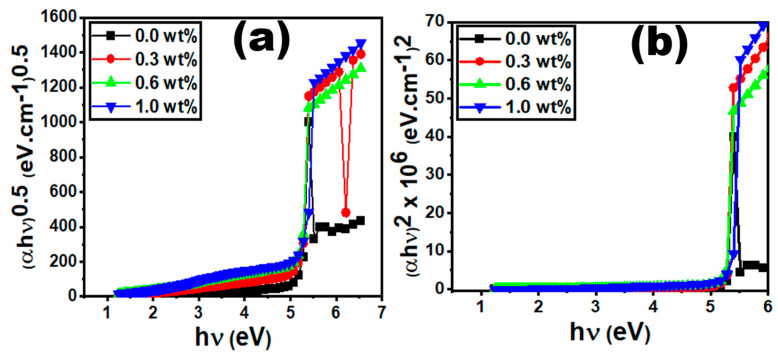
Graphs of (**a**) (αhυ)^0.5^ against hυ and (**b**) (αhυ)^2^ against hυ for the PVC/PVP/g-C_3_N_4_ nanocomposite films.

The vertical excitation energy from the ground state to the first dipole-allowed excited state corresponds to the optical energy gap, which is the lowest energy transition identified in the experimental absorption spectra [42]. We have estimated the optical band gap (E_opt_) from the variation in (αhυ) vs. photon energy (*h*υ) via the Tauc formula [43,44];

(3) $$\alpha h\upsilon =k{\left(hv-E_{g}\right)}^{x}$$

where *k* is a constant. The direct E_dir_ and indirect E_ind_ band gap for allowed transitions are represented by two values: x = 2 and x = 0.5, respectively. To estimate the optical band gap energy E_opt_, we have extrapolated the linear part at zero photon absorption, as represented in Figure 7a,b. The values of the E_dir_ and E_ind_ are reduced for 0.3 and 0.6 wt%, while they are improved for 1 wt%. The decrease in band gap corresponds to the formation of new energy levels between the valence and conduction band.

**Figure 8:**
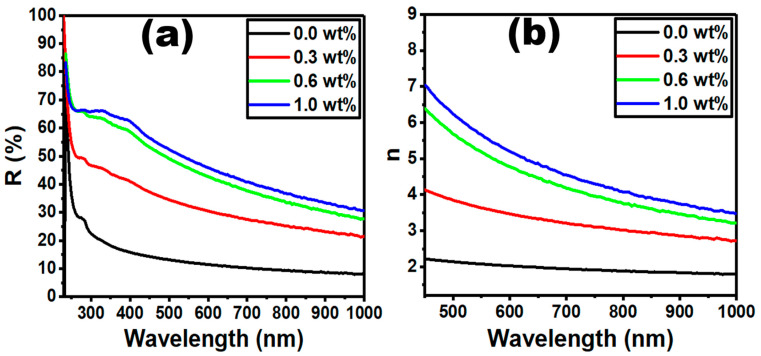
Variation in (**a**) reflectance against wavelength, (**b**) refractive index against wavelength for the PVC/PVP/g-C_3_N_4_ nanocomposite films.

Figure 8a shows the variation in optical reflectance (*R*) vs. wavelength for the PVC/PVP/g-C_3_N_4_ blend matrix at different g-C_3_N_4_ concentrations. The values of *R* enhanced when we added 1.0 wt% of g-C_3_N_4_, while they reduced for 0.3 and 0.6 wt% of g-C_3_N_4_, respectively. We also estimated the refractive index (*n*) from the optical reflectance shown in Figure 8a. To calculate the *n*, we use the following Equation [45]:

(4) $$n=\left(\frac{1+R}{1-R}\right)+\sqrt{\frac{4R}{{\left(1-R\right)}^{2}}-k^{2}}$$

where k represents the extinction coefficient, and it is determined from *k*=αλ/4π. Figure 8b shows that the estimated values of *n* increased when we doped different g-C_3_N_4_ concentrations (0.3, 0.6, and 1.0 wt%) to the PVC/PVP polymer.

The integration of g-C_3_N_4_ nanosheets enhanced the refractive index (*n*), as shown in Figure 8b. This increase resulted from the higher absorption of g-C_3_N_4_ in the visible light range [14]. Many applications, such as optical sensors, solar cells [46,47], emissive displays, and light emitting diodes (LEDs) [48] require materials with a refractive index ≥ 1.65 [49]. As the present PVC/PVP/g-C_3_N_4_ films have a refractive index 1.83–3.96, they are therefore suitable for optical sensors, LEDs, and cladding in optical frequency modulators.

**Figure 9:**
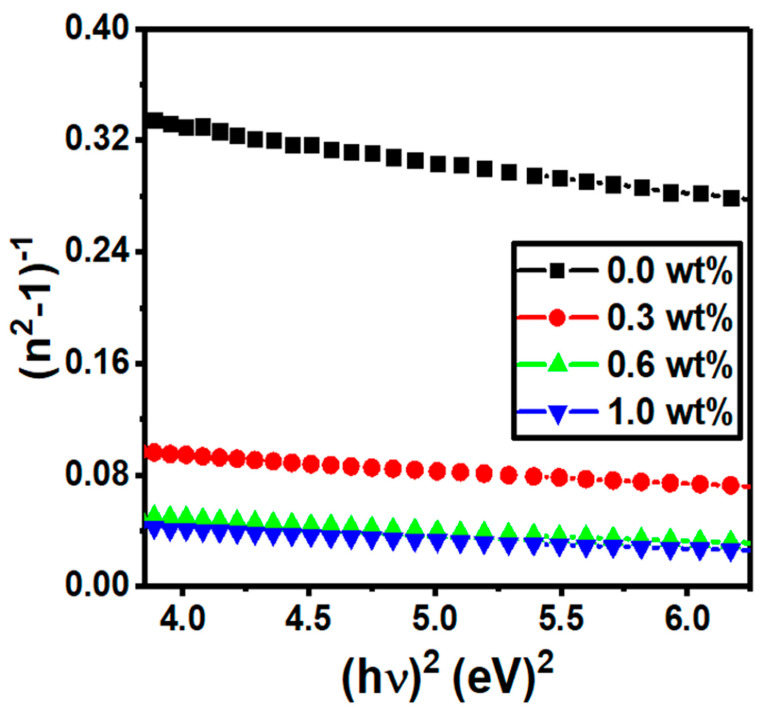
Variation in (n^2^ − 1)^−1^ against ${h\upsilon }^{2}$ for the PVC/PVP/g-C_3_N_4_ nanocomposite films.

Equation (3) represents the refractive index dispersion as a function of photon energy [50]:

(5) $${\left(n^{2}-1\right)}^{-1}=\frac{E_{0}}{E_{d}}-\frac{1}{E_{0}E_{d}}\;{\left(h\upsilon \right)}^{2}$$

where *n*, *E*_0_, *E_d_*, and *h*υ are the refractive index, the single-oscillator energy, dispersion energy, and the energy of the incident photons, respectively [51]. Table 1 represents the values of *E*_0_ and *E_d_* which are calculated from the slope and the intercept of the plots in Figure 9. The *E*_0_ values decrease from 4.21 to 3.14 eV with an increase in the g-C_3_N_4_ concentrations (0.0–1.0 wt%), while the *E_d_* values enhance (9.88–46.08 eV) with the increase in g-C_3_N_4_.

From Equation (4), the static refractive index (*n*_0_) is evaluated at hv→0  as follows [47]:

(6) $$n_{0}{}^{2}=\left(1+\frac{E_{d}}{E_{0}}\right)$$

The refractive index values are enhanced when we add different ratios of g-C_3_N_4_, as listed in Table 1. The estimated values of *n*_0_ are 1.83, 2.90, 3.67, and 3.96 for 0.0, 0.3, 0.6, and 1.0 wt%, respectively.

The oscillator strength (*f*) is presented as follows [52]:

(7) $$f=E_{d}E_{0}$$

where *E_d_* is the dispersion energy, and *E*_0_ is the single oscillator energy. Table 1 shows the *f* values improved when different g-C_3_N_4_ concentrations increased.

Nonlinear effects result from high-intensity light propagating across a material. The most basic of these is the Kerr effect, which is defined as a change in the refractive index in proportion to the optical intensity. Moreover, the nonlinear optical parameters such as linear optical susceptibility ($\chi^{\left(1\right)}$) and the third-order nonlinear optical susceptibility ($x^{\left(3\right)}$) were analyzed for the PVC/PVP/g-C_3_N_4_ nanocomposite films. The estimations of $\chi^{\left(1\right)}$ and $x^{\left(3\right)}$ were completed via the following relations [53]:

(8) $$\chi^{\left(1\right)}=\frac{E_{d}/E_{0}}{4\pi };\;\;x^{\left(3\right)}=6.82\times 10^{-15}{\left(E_{d}/E_{0}\right)}^{4}$$

The data calculations are listed in Table 2 and showed higher values of $\chi^{\left(1\right)}$ and $x^{\left(3\right)}$ for the PVC/PVP/g-C_3_N_4_ films concerning the pure PVC/PVP. The enhanced optical susceptibility performance of these nanocomposite films after the addition of g-C_3_N_4_ nanosheet increase the applicability in optoelectronic devices.

The data of the nonlinear refractive index (n_2_) are estimated depending on the third-order nonlinear optical susceptibility ($x^{\left(3\right)}$) as follows [14,49]:

(9) $$n_{2}=\frac{12\pi x^{\left(3\right)}}{n_{0}}$$

The values of n_2_ recorded in Table 2 show a large increase with an increase in the content of the g-C_3_N_4_ nanosheet. These data of n_2_ for the present PVC/PVP/g-C_3_N_4_ are higher than those listed in the literature [54,55,56].

**Figure 10:**
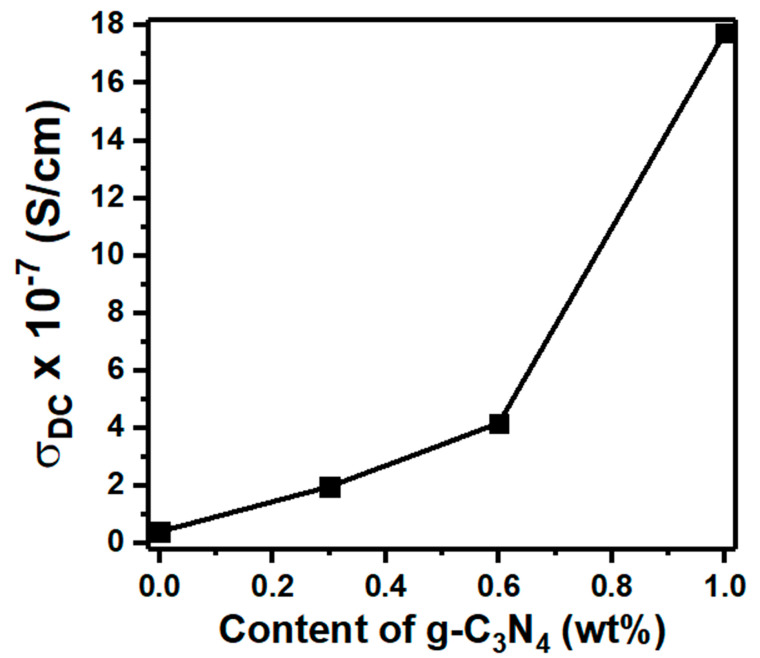
Variation in DC conductivity against g-C_3_N_4_ content for the PVC/PVP nanocomposite films.

Many factors influence the electrical conductivity of materials. The nature of the materials, their dimensions, the temperature, and frequency of electrical fields are the main factors affecting electrical conductivity. Moreover, the polymer–nanofiller interface contributes to the variation in electrical conductivity. The DC conductivity (σ_DC_) of the PVC/PVP/g-C_3_N_4_ films was measured at 300 K and is plotted in Figure 10. When g-C_3_N_4_ was added up to 1.0 wt%, the DC conductivity improved from 4.21 × 10^−8^ to 1.78 × 10^−6^ S/cm. These findings indicate that the g-C_3_N_4_ nanosheet contributes more free ions and ion pairs to the PVC/PVP polymer blend. Higher charge carriers are observed in the polymer film containing 1.0 wt% of g-C_3_N_4_ due to the presence of more π-π∗ bonds. The reduced band gap in the g-C_3_N_4_ nanosheet accounts for the increased conductivity [57].

## 4. Conclusions

The current work investigated the preparation, structural, and optical properties of g-C_3_N_4_ integrated PVC/PVP blend nanocomposites. The data of XRD showed two diffuse peaks at 18.0° and 24.0° in the spectra of blend films which correspond to a semicrystalline structure. FTIR and Raman spectra showed that the g-C_3_N_4_ filling-induced structural modification of the polymeric blend chains significantly affects the physical properties of the g-C_3_N_4_-filled polymeric nanocomposites. From ESEM micrographs, all the polymer films showed homogeneous surface morphologies and strong interconnections between g-C_3_N_4_ and the PVC/PVP blend network. The thermal stability of the PVC/PVP blend nanocomposites improved with the inclusion of g-C_3_N_4_ nanosheets. The refractive index showed higher values (1.83–3.96) after the addition of g-C_3_N_4_. Moreover, the optical band gaps for the PVC/PVP/g-C_3_N_4_ blend films changed with the integration of g-C_3_N_4_ nanosheets. Finally, the data of the nonlinear refractive index (n_2_) and the third-order nonlinear optical susceptibility ($x^{\left(3\right)}$) for these nanocomposite films showed higher performance and thus are suitable for optoelectronic frequency modulators.

## Figures and Tables

**Table 1:** Variation in optical parameters vs. g-C_3_N_4_ concentration for the PVC/PVP/g-C_3_N_4_ blend nanocomposites.

g-C_3_N_4_ Content (wt%)	E_dir_ (eV)	E_ind_ (eV)	E_0_ (eV)	E_d_ (eV)	n_0_	f (eV^2^)
0.0	5.29	5.27	4.21	9.88	1.83	41.56
0.3	5.25	5.23	3.62	26.79	2.90	96.90
0.6	5.25	5.23	3.16	39.41	3.67	124.69
1	5.37	5.34	3.14	46.08	3.96	144.51

**Table 2:** Nonlinear optical parameters for PVC/PVP/g-C_3_N_4_ nanocomposites.

g-C_3_N_4_ (wt%)	χ^(1)^ (esu)	χ^(3)^ × 10^−13^ (esu)	n_2_ × 10^−12^ (esu)
0.0	0.19	2.07	4.26
0.3	0.59	204.6	266.1
0.6	0.99	1650	1695
1.0	1.17	3163	3012

## Data Availability

Data available on request from the corresponding author.
